# Acute Effects Using Light-Emitting Diode Therapy (LEDT) for Muscle Function during Isometric Exercise in Asthma Patients: A Pilot Study

**DOI:** 10.1155/2019/7501870

**Published:** 2019-01-16

**Authors:** Ivan Peres Costa, Fabiano Politti, Lawrence P. Cahalin, Etiene Farah Teixeira de Carvalho, Dirceu Costa, João Carlos Ferrari Corrêa, Rodolfo P. Vieira, Manoel Carneiro Oliveira-Junior, Kátia De Angelis, Roberto Stirbulov, Simone Dal Corso, Paulo de Tarso de Carvalho, Nivaldo Antonio Parizotto, Ross Arena, LucianaMaria Malosá Sampaio

**Affiliations:** ^1^Post-Graduate Program in Rehabilitation Sciences, Nove de Julho University (UNINOVE), São Paulo, SP, Brazil; ^2^Department of Physical Therapy, University of Miami (UM), Miami, FL, USA; ^3^Universidade Brasil, Post-Graduation Program in Bioengineering and Biomedical Engineering, São Paulo, Brazil; ^4^Federal University of São Paulo (UNIFESP), Post-Graduation Program in Sciences in Human Movement and Rehabilitation, Santos, Brazil; ^5^Brazilian Institute of Teaching and Research in Pulmonary and Exercise Immunology (IBEPIPE), São José dos Campos, Brazil; ^6^Santa Casa de São Paulo School of Medicine, São Paulo, SP, Brazil; ^7^Department of Physiotherapy, Laboratory of Electrothermophototherapy, Federal University of São Carlos (UFSCAR), São Paulo, Brazil; ^8^Post-Graduation Program in Biotechnology, University of Araraquara, Brazil; ^9^Department of Physical Therapy and Integrative Physiology Laboratory, College of Applied Health Sciences, University of Illinois, Chicago, USA

## Abstract

The aim of this study was to evaluate the effectiveness of acute application of LEDT in improving peripheral muscle performance during isometric exercise in patients with asthma. Eleven patients, with a mean age 38 ± 10, underwent a single LEDT and sham application in the femoral quadriceps' dominant member (cluster with 50 LED *λ* = 850 nm, 50 mW, 15 s; 37.5 J), 48 h apart in a randomized crossover design. Before and after LEDT and sham application, the patients were submitted an isometric endurance test (60% of the maximum isometric voluntary contraction), up to the limit of tolerance simultaneous recording of surface electromyography. There were no statistically significant differences between groups at the time of contraction (before 41±14 versus 44±16; after 46±12 versus 45±20 s) during the isometric contraction test and inflammatory markers before and after a single LEDT application. A single application of LEDT in the parameters and dose according to the equipment used in the study were not able to promote differences in the time of contraction and the fatigue response in asthmatic patients. However, the chronic effects of LEDT application for improving muscle performance in these patients are unknown and may present different responses during applications for a long time.

## 1. Introduction

Researchers reported acute respiratory myopathies caused by the use of high dosages of systemic steroids [1], but the chronic muscle consequences of prolonged use in asthma remain controversial. Despite the weakness of the respiratory muscles caused by systemic corticosteroids, the peripheral muscles disorders are not well elucidated in these patients. [2]

Pharmacologic therapy can achieve the clinical management of asthma; however an alternative during treatment is the pulmonary rehabilitation [3], to be nonpharmacologic therapy of greater impact in improving physical exercise tolerance, being composed of aerobic training, resistance, or combined training demonstrating positive effects on the improvement of symptoms such as dyspnea and fatigue, increasing strength and endurance in patients with pulmonary diseases. [4, 5]

In two recent studies, Miranda et al. [2014] used a single LEDT application and a combination of super-pulsed laser and light-emitting diodes in patients with chronic obstructive pulmonary disease (COPD) during a maximum isometric exercise protocol, submaximal, and dynamic exercise. The results presented that the local LEDT irradiation of the quadriceps muscle could delay the development of peripheral fatigue during isometric and dynamic exercise and the photobiomodulation with combination of super-pulsed laser and light-emitting diodes is beneficial in improvement of muscular performance, dyspnea, and fatigue sensation in those patients with COPD. [6]

Electrophysical agents such as low level laser therapy (LLLT) and light-emitting diode therapy (LEDT) have been commonly used to treat muscle fatigue and injuries and to promote a better muscle performance when applied to muscles immediately before or after high intensity exercise. A previous study has reported that using LLLT can delay the onset of fatigue and minimize the oxidative stress [8] and its application before high intensity exercise can increase the removal of blood lactate and reduce muscle damage providing rapid muscle recovery [9]. LEDT device has been presented as an alternative therapy using LLLT because of its smaller size and cost [10]. The therapeutic effects presented by this type of laser are (I) analgesic and anti-inflammatory [11], (II) nerve regeneration [12], (III) wound healing [13], and (IV) recovery of muscle fatigue [8].

Based on previously published studies, photobiomodulation can be a novel and noninvasive treatment for muscle fatigue induced by exercise in healthy athletes and can be used in pulmonary diseases such as, for example, COPD and asthma. Therefore, the objective of this pilot study was to evaluate the acute effects of a single LEDT application on muscle function during isometric exercise in patients with asthma.

## 2. Methods

### 2.1. Patients

Subjects with moderate to severe asthma of both sexes, under medical outpatient treatment (six months before participation), clinically stable for three months and under optimized drug therapy, were selected for the study. Subjects were included based on the clinical diagnosis of asthma, which was previously performed by a licensed pulmonologist. In addition, the patients were sedentary, with ages ranging from 30 to 50 years and had oxygen saturation levels ≥ 92% at rest in ambient air. Exclusion criteria included musculoskeletal disease that impaired the performance of the tests, ischemic heart disease, menopause, and rheumatic diseases.

All procedures and exponential risks were reported in detail to all patients before starting the study. In addition, they were informed about the actual research objectives and all participants signed the informed consent form at the beginning of the study. All tests conducted in this study were approved in accordance with the ethical standards of the Institutional Research Ethics Committee (case number: 55844416.6.0000.5511) and with a 1964 Helsinki Declaration and its comparable ethical instants or standards.

### 2.2. Study Design and Protocol

**Figure 1:**
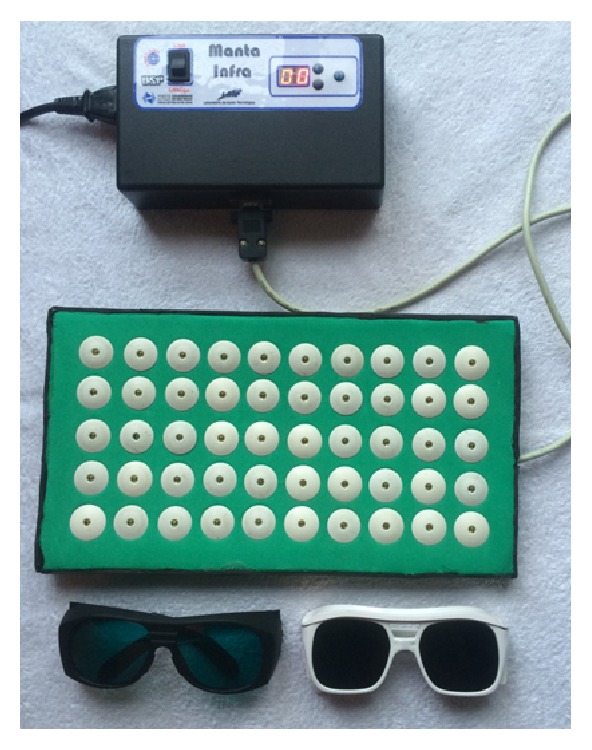
Infrared LEDT containing 50 LEDs (850±20 nm) specially built for research by the Federal University of São Carlos and University of São Paulo. This figure shows the LED board used in the protocol. The same one used by Ferraresi [7].

This is a crossover study. The experiments were carried out in a climatically controlled room at 22-24°C and relative air humidity at 50-60% and performed on different days separated by a 72-hour interval. The protocol was created on the basis of initial assessment (medical history), pulmonary function, body composition [body mass index (BMI), and tetrapolar bioimpedance], the asthma control questionnaire, International Physical Activity Questionnaire (IPAQ) short form, maximum voluntary isometric contraction (MVIC) and the submaximal isometric contraction in the femoral quadriceps dominant leg and collected a blood sample (workflow of the study procedures Figure 1).

### 2.3. Procedures

#### 2.3.1. First Assessment

During the first assessment, information about identification, clinical history, associated diseases, and medications in use were collected in an evaluation record. This procedure was performed by the pulmonologist responsible for the asthma clinic, with the goal of identifying comorbidities that could hinder physical activity engagement during the tests.

### 2.4. Spirometry

The lung function test was performed following the 2002 Spirometry Guidelines and involved the determination of forced vital capacity (FVC), forced expiratory volume in the first second (FEV_1_), and the FEV_1_/FVC, with a calibrated KoKo ® PFT spirometer, adopting the reference values for the Brazilian population [14].

### 2.5. Body Composition

All participants were evaluated individually in the afternoon to avoid the influence of circadian changes. Height, weight, muscle mass, and fat mass were determined. Tetrapolar bioimpedance was measured using the (Biodynamics model 310e, USA) with electrodes on the extremities of the right upper and lower limbs according to the manufacturer's recommendations.

### 2.6. Evaluation of Asthma Control

The Asthma Control Questionnaire (ACQ-6) was used for the evaluation of asthma control. This questionnaire has been translated and validated by Leite et al. [15] and is a valid assessment tool for measuring asthma control among adult outpatients. The ACQ-6 is composed of six items addressing symptoms (nighttime symptoms, daytime symptoms, limitations to activities of daily living, shortness of breath, and wheezing) and the use of a beta-agonist. Score ranges from 0 (no impairment) to six (maximum impairment) points. A score of ≥ 1.5 indicates an 88% chance of clinically uncontrolled asthma.

### 2.7. Physical Activity Questionnaire

The physical activity level was evaluated using the International Physical Activity Questionnaire (IPAQ) short version, translated, and validated in Brazil by Matsudo et al. and its use is indicated for sedentary adult populations (15-69 years of age). The IPAQ scoring protocol provides categorical (insufficiently active, sufficiently active, and very active) and continuous results [expresses the relationship between energy expenditure of physical activity and time in metabolic equivalents (MET), minutes/week].

### 2.8. Skeletal Muscle Function Assessment

The electromyographic signal (sEMG) of the rectus femoris and vastus lateralis was recorded by an acquisition system with 8 channels (EMG800C; EMG System Ltda®, São José dos Campos, Brazil), composed by a conversion board A/D (analog-digital) with 16-bit resolution, bipolar active electrodes with 20 times amplification gain, analog bandpass filter 20-500 Hz, and common mode rejection of 120 dB, sampling frequency of 1 kHz. One of the channels' device is enabled using a load cell (EMG800C; EMG System Ltda®, São José dos Campos, Brazil).

Circular electrodes self-adhesive assets silver chloride (Ag/AgCl), with a diameter of 10 mm (*Medical Trace®*) and interelectrode center distance 20 mm center, were used to collect the sEMG. Each electrode, after cleaning the skin with alcohol, was positioned as follows: (i) rectus femoris: midpoint of a line between the anterior superior iliac spine and the edge of the base of the patella; (ii) the vastus lateralis muscle: the electrodes are arranged from a demarcation line between the iliac spine anterior superior to the lateral border of the patella, to 2/3 of the iliac spine [16]. The ground electrode was fixed to the head of the fibula, ipsilateral to the analyzed member.

For data collection the volunteer was previously instructed to remain seated in a leg flexion with the fixed foot to an arm of the equipment that allowed positioning the knee at an angle of 60% of flexion. Once properly positioned, the volunteer was oriented to perform knee extension against leg extension chair in a maximum isometric voluntary contraction (MIVC). The force generated during this static contraction was obtained by a load cell of appropriately adapted to the arm of the leg extension chair. This procedure was performed three times with collection time of 5 seconds and the range of 3-minute rest. The highest value collected was considered as 100% of the MIVC [17].

After 5 minutes of rest, the volunteers were instructed to conduct a contraction sustained up to exhaustion, with visual feedback provided by a previously established training line, with 60% by MIVC. We followed the methods of Ramos et al. (2015)[18]. Before and after the endurance tests, perception of effort (dyspnea and leg fatigue) was assessed using the modified Borg scale [19].

### 2.9. Light-Emitting Diode Therapy (LEDT) Placebo Application and Therapy

**Figure 2:**
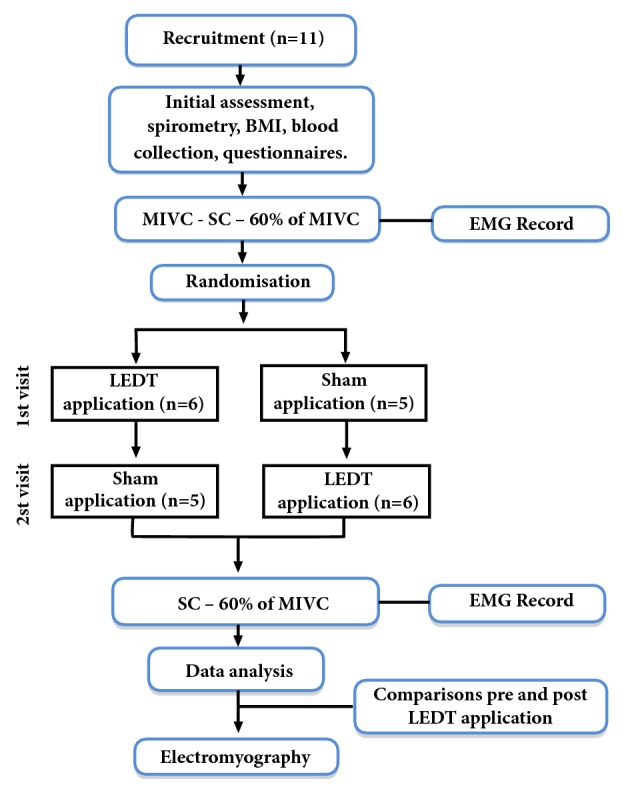
Workflow of the study procedures.

Near-infrared LEDT was applied with an array of multidiode containing 50 LEDs (850±20 nm) specially built for research by the Federal University of São Carlos and University of São Paulo, Brazil (Figure 2) [7]. All parameters were calibrated using Thorlabs*®* (Dachau, Germany) optical meter model PM100D and photodiode power sensor model S130C. All parameters of LEDT used were based on literature reports [7, 20, 21].

Randomization procedure was performed by a simple drawing of lots (A or B), which determined whether active LEDT (A) or sham (B) would be given at the first session. At the second session, participants were crossed over to receive whichever treatment, A or B, was not given at the first session. Neither subject nor evaluators knew if LEDT was effective or placebo during data collection and analysis. A hidden button for placebo or effective LEDT in the LED device was employed to ensure the double-blind procedures. This button was switched previously without the knowledge of either evaluator or the subject. In addition, this button was switched to ‘‘on” (effective LEDT) or ‘‘off” (Placebo) by the one researcher who just participated in the randomization procedure and has been only responsible for switched the button, having no access to data collection and analysis. As the light therapy used was infrared, nobody could identify if the LEDT was effective or placebo while the time display was on.

LEDT (placebo or effective) was applied on two main muscle groups (A and B) used during the test, as described in Table 2 [7]; this table refers to the technical specifications of the equipment used. All muscle groups received LEDT (placebo or effective) 5 minutes before the test in accordance with randomization procedures [7, 20, 22].

### 2.10. Systemic Inflammatory Markers Assessment and Nitrite (NO2) Concentrations

The systemic inflammatory markers were evaluated in blood plasma using the ELISA technique, using commercial kits from R & D Systems according to manufacturer's recommendations, and were assessed as the levels of pro- and anti-inflammatory cytokines (IL-4, IL-5, IL-10, IL-13, IL-17, IL-23, IL-33, IL-1Ra, and VEGF).

The nitrite content of the samples was analyzed using an ozone-based reductive chemiluminescence assay. For this, 50 *µ*L of plasma samples was injected into a solution of acidified triiodide, purging with nitrogen in line with a gas-phase chemiluminescence NO analyzer (Sievers Model 280 NO Analyzer, Sievers, Boulder, CO, USA). Approximately 8mL of triiodide solution (2 g of potassium iodide and 1.3 g of iodine dissolved in 40mL of water with 140mL of acetic acid) was placed in the purge vessel into which plasma samples were injected. The data were analyzed using the software Origin Lab 6.1 [23].

### 2.11. Statistical Analysis

The Shapiro-Wilk test was used to determine the normality of anthropometric, demographic, and clinical data distribution. Parametric data were expressed as mean and standard deviation. Nonparametric data were expressed as median and interquartile interval.

The evaluation of EMG data distribution was realized by the Shapiro-Wilk test. Analysis of variance (ANOVA) of two factors for repeated measures was used to check the EMG signal considering interactions endurance (baseline, 25-100%), treatment (pre x post LEDT application), and analyzing the endurance time considering group (LEDT versus placebo) and treatment (before x after treatment). For all analyzes, we used the Bonferroni adjustment and testing Tukey post hoc peer comparisons.

Results were considered statistically significant when the p value was ≤ 0.05. All data were analyzed using SPSS 20.0 (SPSS Inc., Chicago, USA).

## 3. Results

Eleven subjects were included in the study.  Table 1 displays the data on age, height, BMI, spirometric values, percentage of lean mass, fat mass IPAQ, medications in use, mean of MIVC and 60% MIVC, and ACQ scores. The protocol was performed safely without any adverse events documented in any subject. According to the IPAQ classification the sample was composed of physically very active individuals (90.90%). The irradiation was performed in contact on skin with an array of multidiode containing 50 LEDs. The features of each LED and parameters of LEDT before the MIVC test are presented in Table 2.

**Figure 3:**
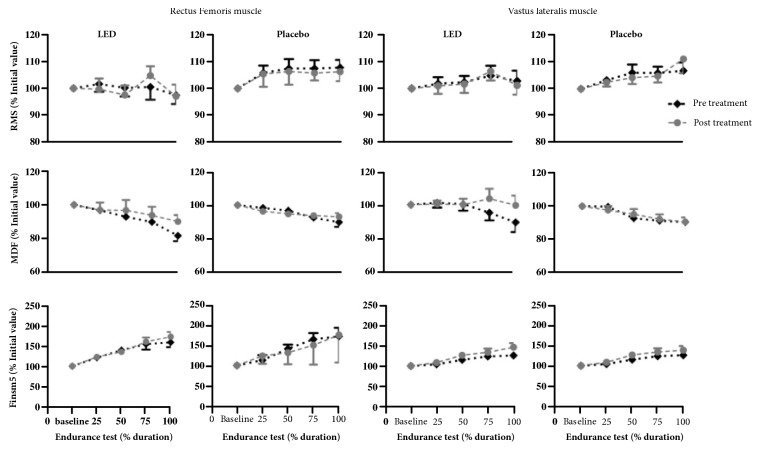
Interactions between sEMG analysis expressed in mean and standard error of the rectus femoris and vastus lateralis.

There were no statistically significant differences between groups at the time of contraction (before 41±14 versus 44±16; after 46±12 versus 45±20 s) during the isometric contraction test before and after a single LEDT application (treatment) versus endurance. Interactions between sEMG analyses are presented in Figure 3.

**Figure 4:**
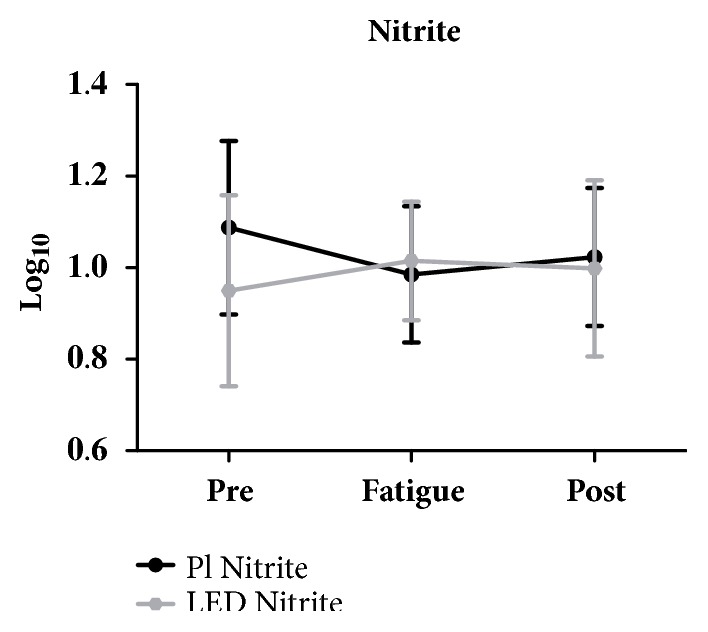
Nitrite's blood level.

**Figure 5:**
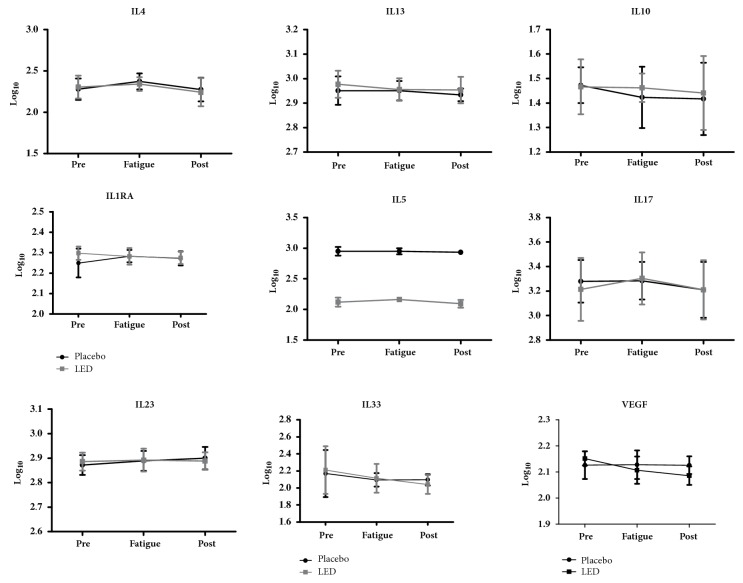
Systemic inflammatory markers blood levels (pro and anti-inflammatory cytokines).

There were no statistically significant differences in blood levels of pro- and anti-inflammatory cytokines in IL-4, IL-5, IL-10, IL-13, IL-17, IL-23, IL-33, IL-1Ra, VEGF, and Nitrite of groups, before and after a single LED application (Figures 4 and 5).

## 4. Discussion

This is the first study to assess the acute effects of LEDT for muscle function of isometric exercise and systemic inflammatory markers in patients with moderate to severe asthma. The main finding of this study did not find statistically significant difference in these variables evaluated after a single application of LEDT.

Previous studies have shown that photobiomodulation administered in peripheral muscles immediately after endurance exercise can improve contractile function, prevent muscle damage caused by exercise, and improve recovery postworkout muscle strength [22]. Following this information, we investigated whether the same effects found in athletes [24] and healthy individuals [25] would be found in patients with moderate to severe asthma.

In their study Vieira et al. [26] found increased muscle performance in isokinetic dynamometer in the extensor muscles of the knee at forty-five healthy women after use of phototherapy. The authors suggest that the endurance training associated with phototherapy was able to reduce muscle fatigue.

Miranda et al. [6] were the first to investigate the LEDT acute effects in COPD patients; a smaller drop in median frequency (MDF) was found after the sustained isometric contraction protocol, with consequent increase in the endurance test time isometric muscle in ten COPD patients (FEV_1_ 50 ± 13% of predicted) after a LEDT application compared to placebo application in the quadriceps muscle.

Clinical studies that investigated the phototherapy acute effects applied immediately before exercise in young athletes found the increase in the number of muscle contractions and lower serum levels of blood lactate, C-reactive protein, and Creatinine Kinase (CK), indicating an increase in fatigue resistance and accelerated postexercise recovery [8]. In the present study, we did not evaluate the serum levels of these markers, but there was no statistical difference between the pro- and anti-inflammatory systemic cytokines and oxidative stress assessed by nitrite.

The microcirculation increased after phototherapy irradiation is due to the release of nitric oxide (NO) into the bloodstream. A recent study showed increased levels of NO venous blood fifteen healthy subjects after the phototherapy application [27]. In the present study we do not evaluate the serum concentration of NO, but by assessment of serum nitrite levels there was no statistically significant difference between the comparison in rest, after fatigue testing, and a single LEDT application.

Our population was composed of physically active individuals according to the IPAQ classification, results that corroborate the findings in the literature. Some studies have shown that physical exercise promotes an increase in the concentrations of NO and oxidative stress. Jacomini et al. [28] after evaluating the relationship between blood pressure, nitric oxide, and oxidative stress in a group of physically active elderly women found a low prooxidant and antioxidant high. In another study Gomes et al. [29] found no difference in plasma nitrite after 3 months of exercise in patients with metabolic syndrome. We can infer the fact that our sample was classified as very active; we would not find differences in plasma concentrations of nitrite.

In another study that evaluated the acute effects of exercise associated with the reduction of nitric oxide exhaled in asthmatic adults, Scott et al. [30] compared physically active and inactive individuals evaluating serum concentrations of IL-6 IL-10, and IL-1RA interleukins, and, as a result, there was no statistically significant difference in the physically active group. Although the study is an aerobic exercise protocol, such results corroborate our study in acute responses after resistance exercise protocol, which did not found statistically significant differences in anti-inflammatory interleukins IL-10 and IL-1RA at rest, after a single LEDT application, and immediately after the fatigue test. In addition, similarly to the study from Scott et al. [30] we also have not found changes in the levels of proinflammatory cytokine IL-6. Furthermore, the values for other proinflammatory Th2 cytokines (IL-4, IL-5, and IL-13), which are the central cytokines involved in asthmatic allergic response, also were not changed after LEDT. Similarly, IL-17 and IL-23, which are considered proinflammatory cytokines involved in the exacerbation of proinflammatory responses, also were not changed by LEDT. IL-33 is now being considered a proinflammatory cytokine centrally involved in asthma pathogenesis, which also has not been changed after LEDT. VEGF is a proangiogenic growth factor which is upregulated in asthma. In the present study, no differences were found in the VEGF levels after LEDT. Concerning the anti-inflammatory cytokines (IL-1ra and IL-10), the present study showed that a single LEDT application did not result in any changes in the levels of these anti-inflammatory cytokines.

Ramos et al. [18] in their study evaluated functional capacity by the incremental shuttle walk test, IPAQ, and 1RM of asthmatic individuals compared to a healthy individuals' group. As a final result, it could be observed that asthmatic individuals presented reduced functional capacity, reduced physical activity levels, and a higher percentage of body fat when compared to the healthy individuals group. This suggests that such patients have a sedentary lifestyle due to poor physical performance. In addition to physical inactivity, the use of high doses of medications such as glucocorticoids and *β*2-agonists (used to treat some respiratory diseases) can cause atrophy and loss of muscle strength. These may be more evident in unusual cases, where the onset of such a process is acute. However, muscle impairment is typically slow onset and insidious, manifesting itself by muscle weakness and fatigue while performing tasks such as walking.

Frequently the search for adjuvant therapies to gain strength and endurance has been applied in the clinical practice of pulmonary rehabilitation. Equipment such as LEDT is presented as an alternative to gain strength and endurance according to studies in healthy individuals, athletes, and the previous study in carrier COPD population, delaying the development of fatigue in peripheral muscles. Before your application, it is essential to know the type of tool to be used and the appropriate dose for the help in gaining strength and decreased fatigue.

The literature has reported the possible biphasic dose-response [31] that has been used to explain the findings obtained with photobiomodulation in experimental studies and clinical trials. This biphasic dose-response reports that light fluencies (J/cm2) using the doses 0.03, 0.3, and 3 J/cm2) produced significant increments in ATP (adenosine tri-phosphate) synthesis and MMP (mitochondrial membrane potential) activity with peak at 3 J/cm2. Therefore, this behavior of the photobiomodulation offers the plausible one explanation of the results found in previous studies that used very different light doses (energy J) [32, 33]. De Marchi et al. in his study with healthy subjects applied the quadriceps muscle and 180J observed positive results related to performance and muscle fatigue. Miranda et al. 2016 applied 270J in quadriceps and also observed positive results in fatigue and muscle performance during the cardiopulmonary test in sedentary men. Our results corroborate with the data found for da Silva Alves et al. applied in the quadriceps 42 J in healthy individuals, finding no positive results on the performance and muscle fatigue. We can take into consideration some aspects like exposure time the boots, the dose of application per muscle group, the number of points to be applied, and the fact that the patients present systemic repercussions. In a systematic review of Borsa et al. 2013 [22] a therapeutic window of 8.4 the 417 J was observed, so we can say that our study is within that range with 75J. But, we did not know what kind of response pathophysiological patients with asthma might have.

According to Leal-Junior et al. (2015) [34], nonsignificant results can be explained by a low dose or area of irradiation, resulting in insufficient irradiation. A single application in peripheral muscle has no effect on the strength gain and decreased fatigue, but the chronic effects of LEDT application and what the ideal dose to be established for its use are still unknown in the asthmatic population, necessitating studies to enhance its use as an adjunct feature during pulmonary rehabilitation.

## 5. Conclusion

In this study, the acute LEDT applications did not differ in the time of fatigue response to asthma patients. However, the chronic effects of LEDT application for improving muscle performance in these patients are unknown and may present different responses during applications a long time.

## Figures and Tables

**Table 1:** Anthropometric, demographic, and clinical.

	Asthmatic Individuals n=(11)
Age (years)	38.81 ± 11.34
Gender (M/F)	1/10
Heigth (m)	1.62 ± 0.08
Weigth (Kg)	69.22 ± 12.17
BMI (Kg/m^2^)	26.29 ± 4.84
FCV (%)	75.55 ± 20.06
FEV1 (%predict)	71.44 ± 17.86
Muscle Mass (%)	63.81 ± 13.05
Fat Mass (%)	34.27 ± 11.83
MIVC (Kg)	25.20 ± 11.15
60% MIVC (Kg)	14.19 ± 6.24

ACQ	1.55 [1.17- 2.75]
**(IPAQ), (**%**)**	
Inactive	-
Moderate Active	9.09%
Very active	90.90%
**Medication (**%**)**	
long duration *β*-2 agonists	100%

Data reported as mean ± standard deviation or median (interquartile intervals). Legend: BMI = body mass index; LBW = lean body weight; FEV_1_= forced expiratory volume in the first second; ACQ = Asthma Control Questionnaire; IPAQ= International Physical Activity Questionnaire (according to the IPAQ classification).

**Table 2:** Parameters of LEDT and regions of irradiation on leg before MIVC test.

Number of LED's: 50;
Wavelength: 850 ± 20 nm (infrared);
Frequency: Continuous output;
Optical output: 50 mW per/diode LED;
LED spot size: 0.2 cm^2^;
Power density: 250 mW/cm^2^;
Treatment time over each muscle group: 15 s;
Energy per diode at 15 s: 0.75 J;
Energy density per diode at 15 s: 3.75 J/cm^2^;
Number of irradiation points per muscle group: 50;
Total energy delivered per muscle group: 37.5 J;
Muscle group irradiated before fatigue test: Femoral quadriceps of the dominant member and hamstrings of the dominant member;
Total energy delivered on body: 75 J;
Total power output: 2500 mW;
Application mode: device held coupled in skin contact.

J= Joule; mW= micro-Watts W= Watts; cm= centimeters; nm= nanometers; m= meters. This table refers to the technical specifications of the equipment used. This same information is contained in the publication of Ferraresi [7].

## Data Availability

The data used to support the findings of this study are available from the corresponding author upon request.
